# Linke Außenpolitik – einige Überlegungen

**DOI:** 10.1007/s12399-021-00851-y

**Published:** 2021-05-19

**Authors:** Dietmar Bartsch

**Affiliations:** Fraktion DIE LINKE im Deutschen Bundestag, Platz der Republik 1, 11011 Berlin, Deutschland

**Keywords:** Völkerrecht, Kooperation, Krisengebiete, Staatenorganisationen, NATO, International law, Cooperation, Areas of crisis, Organizations of states, NATO

## Abstract

Im Zentrum der außenpolitischen Agenda der Partei DIE LINKE steht der Gedanke, dass die kooperative Bearbeitung von Interessenskonflikten und globalen Problemen eine stabile Völkerrechtsordnung voraussetzt. Der vorliegende Beitrag diskutiert daher ausgewählte Staatenorganisationen bezüglich ihrer Eignung für eine kooperative Praxis.

## Abstract (en)

At the heart of the foreign policy agenda of the party DIE LINKE is the idea that the cooperative handling of conflicts of interest and global problems requires a stable international legal order. This article therefore discusses different organizations of respective states and their aptitude for cooperation.

## Einführung

Ich könnte es mir einfach machen und die einschlägigen Passagen des letzten Wahlprogramms der LINKEN in ein wenig Prosa übersetzen. Bis auf ein paar Updates ist nicht zu erwarten, dass sich im neuen Wahlprogramm der LINKEN Grundlegendes bezüglich der außenpolitischen Vorstellungen der LINKEN ändern wird, obwohl das vielleicht hier und da notwendig wäre. Jedoch möchte ich einige Überlegungen anstellen, die verdeutlichen sollen, was eigentlich hinter den programmatisch formulierten Positionen steht.

## Der normative Rahmen der internationalen Politik

Internationale Politik findet zwar nach wie vor als Interessenpolitik statt, der Begriff der Wertegemeinschaft verschleiert dies nur. Aber selbst wenn man jedes Interesse als gleich legitim betrachten würde, gibt es weitere Bewertungsmöglichkeiten der Außenpolitik von Staaten. Diese Bewertungsmöglichkeiten sind durch ein Geflecht völkerrechtlicher Verpflichtungen gegeben. Häufig werden Menschenrechte als ein Bewertungsmaßstab benannt. Das ist auch richtig, solange man nicht dem Irrtum unterliegt, dass es sich bei Menschenrechten um etwas substanziell vom Völkerrecht Verschiedenes handelt. Menschenrechte haben gerade durch völkerrechtliche Kodifizierung Geltungskraft. Sie stehen nicht neben oder gar über dem Völkerrecht. Dennoch wird häufig davon ausgegangen, dass ein Konflikt zwischen Menschenrechten auf der einen Seite und Völkerrecht auf der anderen Seite existiere. Das war in den deutschen Diskussionen vor allem um den Kosovokrieg, aber auch um den Afghanistankrieg, deutlich nachweisbar. Auf diese Weise wird nicht nur suggeriert, dass durch Kriege die Menschenrechtslage spürbar verbessert werden könne. Es wird auch Schaden am Völkerrechtsdenken angerichtet. In einer an Carl Schmitt erinnernden Manier wird zur *Entscheidung* genötigt zwischen den *Werten* und dem *bloß Formalen* der Völkerrechtsordnung. Nur dadurch, dass viele politische Akteure und Kommentatoren diese Ideologie wirklich glauben, ist es auch erklärbar, dass diese die Abtrennung der Krim von der Ukraine und ihren anschließenden Beitritt zur Russischen Föderation verurteilten, aber einst die Abtrennung des Kosovo von Serbien verteidigten.

Generell ist der Respekt vor dem Völkerrecht schwächer geworden. Der letzte Irakkrieg unter dem damaligen US-Präsidenten George W. Bush zeigte, dass den USA völkerrechtliche Verpflichtungen gleichgültig waren, sie einen offenen Aggressionskrieg führen konnten, auch wenn sie dabei die NATO spalteten. Das damalige politische Konzept des Unilateralismus, das zweifellos auch die Beziehungen zu Russland komplizierter gemacht haben dürfte, fand seine radikalisierte Fortsetzung in *America First*, auch wenn letzteres Konzept dazu eine isolationistische Komponente hat. Unter dem ehemaligen US-Präsidenten Donald Trump bedeutete das einen verantwortungslosen Umgang mit völkerrechtlichen Verpflichtungen. Als besonders schädlich muss man den Ausstieg der USA aus dem Iran-Atom-Abkommen, dem Pariser Klimaabkommen und dem Rüstungskontrollabkommen bezüglich der Intermediate Range Nuclear Forces (INF) ansehen. Auch den Rückzug aus der Weltgesundheitsorganisation kann man gerade angesichts der grassierenden SARS-CoV-2-Pandemie nicht billigen.

## Prinzip: Kooperation statt Konfrontation

Die Partei DIE LINKE sieht eine friedliche Zukunft nur in der Festigung der Autorität des Völkerrechts. Das muss ohne Ausnahmen gelten. Wie sich jüngst beim Konflikt um Nagorny-Karabach zeigte, können sogenannte *Frozen Conflicts* stets in neue bewaffnete Konflikte umschlagen. Gerade auf dem Gebiet der ehemaligen Sowjetunion gibt es etliche derartiger Konfliktzonen. Es wäre denkbar, dass etwa im Rahmen der Organisation für Sicherheit und Zusammenarbeit in Europa (OSZE), in der alle ehemaligen Sowjetrepubliken mitarbeiten, verbindliche Lösungen gefunden werden könnten. Das setzt aber eine kooperative Haltung aller beteiligten Staaten voraus. Spannungen zwischen der NATO und der Russischen Föderation sind dabei gewiss nicht hilfreich.

Es ist auch denkbar, dass völkerrechtliche Modernisierungen geschaffen werden. Wie geht man denn mit Akteuren um, die das Produkt einer Ethnisierung von Konflikten sind? Können sie als Völkerrechtsakteure, mit welchen Einschränkungen auch immer, angesehen werden? In manchen Fällen tun wir dies: bei der Palästinensischen Beifreiungsorganisation (PLO), bei der Polisario und bei den Kurdischen Autonomiegebieten im Irak. Dieses Problem stellt sich sowohl in Europa als auch in den ehemaligen Kolonien europäischer Staaten. Die Herero sind kein Staat und daher kein Völkerrechtssubjekt. Wären sie dies, wären ihre Ansprüche auf Wiedergutmachungsleistungen in Bezug auf den Völkermord an diesem Volk durch das Deutsche Kaiserreich leichter durchsetzbar. Gerade hinsichtlich der Durchsetzung von Wiedergutmachung kolonialen Unrechts erweist sich das Völkerrecht als nur begrenzt geeignet. Zugleich ist die Idee der Aufwertung substaatlicher Akteure nicht unproblematisch, weil es die Gesamtstaatsperspektive der betroffenen Länder nachhaltig verändern kann. Der Irak beispielsweise ist heute gesamtstaatlich sehr geschwächt, er ist zur Beute konkurrierender ethnokonfessioneller Gruppen geworden – auch eine Folge der US-Intervention.

Wenn es ein wirkliches Interesse an Gewalteindämmung und realer Konfliktlösung gibt, muss man den Blick auf obige Fragestellungen und mögliche institutionelle Rahmen lenken. Das ist dann etwas anderes als ein militärischer Interventionismus, an den sich der Westen gewöhnt hat.

Es gibt eine Reihe von Konfliktfeldern, in denen der Westen aktiv werden kann, teilweise auch aktiv ist. Dazu meine Ansichten.

### Naher Osten

Vorweg ist zu betonen, dass DIE LINKE die Haltung der anderen demokratischen Parteien im Bundestag teilt, dass Deutschland zu Israel eine besondere, solidarische Haltung einnehmen muss. Der Staat Israel ist ohne den nationalsozialistischen Völkermord am jüdischen Volk nicht zu denken. Wer für die deutsche Geschichte im Sinn eines „Nie wieder!“ Verantwortung übernehmen will, dem kann Israel, der Staat, der von den Überlebenden des Holocaust aufgebaut wurde, nicht gleichgültig sein.

Israel hat seit seiner Gründung mit Angreifern zu tun, die das Existenzrecht dieses Staates in Zweifel ziehen und offen mit Vernichtung drohen. Das Land wurde mit Kriegen und Terror überzogen. Das macht es verständlich, dass Friedensperspektiven es nicht leicht haben, erst recht nicht, wenn Politiker*innen wie der ehemalige Präsident der USA Trump Illusionen schüren. Grundlage einer möglichen Friedenslösung ist die sogenannte Zwei-Staaten-Lösung mit einem palästinensischen Staat und dem Staat Israel in den Grenzen von 1967. Das ist auch das allgemeine völkerrechtliche Framing. Aus Sicht meiner Partei, und darin tritt auch ein Unterschied zur Bundesregierung und anderen Parteien ans Licht, tut die Bundesregierung allerdings zu wenig, um eine Friedenslösung zu ermöglichen. Dabei ist es nötig, eben auch die palästinensischen Interessen mit in den Blick zu nehmen.

### Iran

Der Iran ringt mit anderen Staaten um die Dominanz im Nahen Osten. Das wird offensichtlich bei der Unterstützung schiitisch geprägter Parteien, Bewegungen und Milizen. Konkurrenten sind die sunnitisch geprägten Staaten Saudi-Arabien und Türkei. Zudem bedroht der Iran Israel, etwa durch seine Unterstützung der Hisbollah oder durch das Atomwaffenprogramm.

Dem Abbau der nuklearen Bedrohung sollte das Atomabkommen dienen. Dieses ist zwar von den USA aufgekündigt worden, nicht aber von allen Vertragsparteien. Ich hoffe, dass die USA zurückkehren werden, um den Zerfallsprozess dieses Abkommens zu stoppen. Denn der Iran lässt deutlich erkennen, dass er sich nicht mehr so stark an das Abkommen gebunden fühlt. Hier ist eine Neubelebung dringend erforderlich.

Gelänge dies, könnte das ein Ausgangspunkt sein, um auch die Unterstützung schiitischer Milizen durch den Iran zum Thema zu machen. Gerade beim Jemen zeigt sich jedoch, dass es neben dem Iran noch andere Staaten gibt, die einen negativen Einfluss auf die Region ausüben: so z. B. Saudi-Arabien. Den Iran zu *bändigen* heißt auch, Saudi-Arabien und seine Rolle zum Thema zu machen. Das jedoch kommt seitens der Bundesregierung deutlich zu kurz. Im Gegenteil! Für Rüstungsgeschäfte ist sie sehr aufgeschlossen, was angesichts der militärischen Aktivitäten Saudi-Arabiens etwa im Jemen äußerst verantwortungslos ist.

### Türkei

Die Türkei ist ein weiterer Akteur, der um regionale Hegemonie ringt und dabei Destabilisierungen in Kauf nimmt. Gegenwärtig ist die Türkei an Konflikten in Libyen, Nagorny-Karabach, Syrien, Griechenland und Zypern direkt oder indirekt beteiligt. In Libyen und Syrien wirkt sich das unmittelbar destabilisierend auf die jeweils innerstaatlichen Verhältnisse aus, die Eskalation des Nagorny-Karabach-Konflikts zum bewaffneten Konflikt zwischen Armenien und Aserbaidschan hat sie aktiv vorangetrieben, auch unter Einsatz von islamistischen Söldnern sowie der Restrukturierung der Kommandostrukturen der aserbaidschanischen Streitkräfte. Im Seerechtsstreit mit Griechenland und Zypern provoziert sie hemmungslos.

Weder die NATO scheint in der Lage oder auch nur Willens zu sein, diesem Treiben ein Ende zu machen, was nichts anderes als Billigung ist, auch wenn es heißt, dass der schwelende Konflikt zwischen den NATO-Staaten Griechenland und Türkei sich zuspitzen könnte. Auch die EU ist hier schweigsam. Das liegt zum einen am EU-Türkei-Deal, um die Fluchtbewegungen in Richtung EU unter Kontrolle zu bringen, das liegt aber auch an divergierenden Interessen unterschiedlicher EU-Staaten in der Mittelmeerregion.

Aus Sicht der LINKEN lassen sich diese Konflikte nicht isoliert betrachten. Sie können nur in einem die gesamte Region betreffenden Ansatz bearbeitet werden. Dabei ist es dringend erforderlich, falsche Rücksichtnahmen zu unterlassen, um sich zumindest eine gewisse Glaubwürdigkeit zu erhalten.

## Herausforderungen

Die Schwierigkeiten, mit denen wir heute zusätzlich konfrontiert sind, werden oft als *Herausforderungen* bezeichnet. Die scheinbare Sportlichkeit dieses Ausdrucks kann in die Irre führen. Es geht um etwas anderes: um ernste Probleme. Manchmal höre ich die Wendung, dass die Welt aus den Fugen geraten sei. Auch ich habe diese Wendung gebraucht. Tatsächlich haben wir es mit einer multiplen Krise zu tun. Seit der Finanzkrise ist das finanzgetriebene System kapitalistischer Akkumulation in eine tiefe Krise geraten. Diese löste die *Eurokrise* aus, die natürlich keine Währungskrise im klassischen Sinn ist, sondern eine politische Krise des Euro. Dann ist der Sozialstaat in einer tiefen Krise und ein Bewusstsein um die ökologische Krise ist längst Alltagsbewusstsein. Zu all dem kommt nun hinzu, dass seit dem Ende des Kalten Kriegs ein Kampf um die globale Neuordnung geführt wird. Die multiple Krise findet ihren Niederschlag auch im Erstarken rechtspopulistischer und rechtsradikaler Parteien und Bewegungen. Sie beuten die Überforderung vieler Menschen durch die scheinbar permanent gewordene Krisenhaftigkeit aus. Da gedeihen dann auch antiwestliches, im Kern aufklärungsfeindliches Ressentiment und Antisemitismus.

In den Neuordnungskonflikten finden wir neben den jeweils regionalen Hegemoniebestrebungen folgende globale Akteure: die russische Föderation, die sich als globaler Akteur neu aufzustellen beginnt, die Volksrepublik China, die ein eigenes, auf Infrastrukturprojekten basierendes, ökonomisches Globalisierungsmodell durchzusetzen beginnt, und die USA, die als *Führungsmacht* des Westens sich um die Situation der frühen 1990er-Jahre gebracht sieht. Damals brachte Francis Fukuyama den Zeitgeist auf den Begriff: Die marktwirtschaftlichen und liberalen Demokratien seien auf dem Vormarsch, die Geschichte sei an ihr Ende gekommen. Aus irgendwelchen Gründen glaubte man im Westen, dass der neoliberale Ausverkauf Russlands immer so weitergehen werde und dass China unbedingt die Werkbank der Welt bleiben wolle und keinerlei weitergehende Ambitionen habe oder je entwickeln wolle. Zuweilen wirken die Reaktionen auf Russlands und Chinas politisches Handeln einfach nur narzistisch. Das Selbstbewusstsein dieser Länder kränkt den Sieger des Kalten Krieges.

Das heißt überhaupt nicht, diese Länder von Kritik freistellen zu wollen. Aber wenn Kritik, dann auf der Grundlage von klaren Kriterien. Da erscheint es dann doch seltsam, wenn man im Seidenstraßenprojekt Chinas ein Sicherheitsrisiko sieht, wie es der Generalinspekteur der Bundeswehr Eberhard Zorn kürzlich in einem Interview tat^1^, zugleich aber nicht akzeptieren will, dass Russland hinsichtlich der europäischen Politik der Östlichen Partnerschaft auf ähnliche Gedanken kommen könnte. Es ist wohl eher umgekehrt. Ökonomische Kooperation stiftet Sicherheit, das war bereits im Kalten Krieg so. Da stimmt es mich optimistisch, dass die Gespräche zwischen der EU und China über eine Vertiefung der wirtschaftlichen Beziehungen zu einem Durchbruch geführt haben. Das ist anscheinend auch der deutschen Bundeskanzlerin Angela Merkel zu verdanken. Denn auch die Zugeständnisse Chinas über das rein Ökonomische hinaus sind von Bedeutung: die Zusage, dem Pariser Klimaschutzabkommen beizutreten und die Konvention der International Labour Organization (ILO) gegen Zwangsarbeit zu ratifizieren.

Im Islamismus, insbesondere in der Bedrohung durch den islamistischen Terror, besteht nach allgemeiner Auffassung ein weiteres großes Problem, das bewältigt werden muss. Dieser Ansicht schließe ich mich an. Jedoch muss man sich eingestehen, dass der Anti-Terror-Krieg, ausgerufen nach dem 11. September 2001, gescheitert ist. Er hat Nebenfolgen, die nur weiteren Terror erzeugen. Nach der Zerschlagung der Taliban-Herrschaft ist Al-Qaida *emigriert.* Übrigens werden irgendwann alle westlichen Truppen aus Afghanistan abgezogen sein. Ob es dann eine politische Lösung ohne Taliban geben wird, ist mehr als fraglich. Ich bin ein Gegner des militärischen Interventionismus. Aber selbst, wenn ich es nicht wäre, würde ich doch fordern, dass man aus solchen *Abenteuern* wie dem Afghanistankrieg etwas lernt. Auch deshalb ist die Forderung der LINKEN, die Bundeswehr aus Afghanistan schnellstmöglich und vollständig abzuziehen, richtig.

Es scheint mir an der Zeit, über den Zusammenhang zwischen Terrorismus und Staatsschwächung nachzudenken. Nach der US-Intervention im Irak trat eine Verfassung in Kraft, die ethnokonfessionelle Gruppen stärkte und den Gesamtstaat schwächte. Insbesondere erfuhren die vormals privilegierten Sunniten Diskriminierung, was es den aus Afghanistan emigrierten Al-Qaida-Kadern erleichterte, Anhänger*innen für terroristische Aktivitäten zu rekrutieren. Aus der irakischen Al-Qaida-Abteilung und ebenfalls verdrängten Anhänger*innen der Baath-Partei, vor allem jedoch baathistischen Militärs, bildete sich eine neue Melange: die künftige ISIS-Formation. Denn durch den Bürgerkrieg in Syrien wurden ebenfalls Gebiete *frei,* in denen diese Formation aktiv werden und sich als neue Ordnungsmacht aufspielen konnte. Ihre Abgrenzung gegen Al-Nusra, dem syrischen Ableger von Al-Quaida, machte die Neuformierung zu etwas von Al-Qaida Unabhängigem erforderlich. Zugleich bildeten sie Kernelemente einer eigenen Staatlichkeit aus: Militär und Verwaltung. Bezüglich Syrien wird der Einfluss des Westens auf den Staatszerfall von traditionell antiimperialistischer Seite überschätzt. Dennoch gab es ihn. Bezüglich des Irak jedoch kann man ihn nicht überschätzen. In Libyen, wo es auch eine Intervention gab, gibt es nun Milizen mehr oder weniger islamistischer Prägung und konkurrierende *Regierungen*. Im durch Bürgerkrieg und die Kriegsführung Saudi-Arabiens zerrütteten Jemen ebenso. Es wäre an der Zeit, Stabilisierungspolitik zu betreiben, auch wenn einem die Machthaber nicht ganz passen. Es ist ohnehin nicht plausibel, weshalb die einen so schlimm sind, dass sie auf jeden Fall weg müssen, während man mit Saudi-Arabien Geschäfte macht.

Spätestens mit dem Islamischen Staat (IS) ist noch etwas deutlich geworden. Der IS besaß und besitzt eine nicht unerhebliche Ausstrahlungskraft auf junge Menschen in den westlichen Gesellschaften. Das sind Menschen, die hier aufgewachsen sind. Was, diese Frage müssen wir uns stellen, versagt in unseren Gesellschaften, dass solche zutiefst reaktionären ideologischen Konzepte wie der Islamismus verfangen und zu terroristischen Handlungen führen können? Die Antwort auf diese Frage liegt nicht bei der Sicherheitspolitik. Deshalb ist es eine sehr wichtige Frage. Auch unsere Gesellschaften weisen offenbar schwere Defizite auf, denen wir begegnen müssen. Eine Mischung aus verstetigter Armut und Islamophobie dürfte dabei eine Rolle spielen.

## NATO

Nach dem Ende des Kalten Krieges steckte die NATO in ihrer größten Legitimationskrise. Die staatssozialistischen Länder unter der Führung der Sowjetunion sowie deren Militärblock, die Mitgliedsstaaten des Warschauer Vertrags, hatten dem Sozialismus abgeschworen, den Warschauer Vertrag aufgelöst, und auch die Sowjetunion begann bereits zu zerfallen. Es blieb nur die NATO übrig und der fehlte der *Feind*. Sicherheit kann man nur versprechen, wenn es Unsicherheit gibt und wenn klar ist, wer diese Unsicherheit erzeugt.

Um ihre Existenz zu sichern, war die Produktion von neuen Feindbildern erforderlich. Da überall auf der Welt immer wieder Unsicherheiten auftreten, die irgendwer schon zu verantworten haben wird, expandierte die NATO gegen eigene Zusagen schnell mal ihren Raum (*Osterweiterung*) und ihren *Verantwortungsbereich*. Letzteres ist dann möglich, wenn man am Sicherheitsbegriff arbeitet. Dieser ist sowohl geographisch als auch inhaltlich erweitert worden. Geographisch: Im Prinzip fühlt sich die NATO überall zuständig, für Ordnung und Sicherheit zu sorgen. Das alte Verständnis, dass die NATO gemeinsam ihre Mitgliedsstaaten vor Aggressionen schützt, ist jedenfalls nicht mehr gültig. So hatte Rest-Jugoslawien keinen NATO-Staat angegriffen (und zu diesem Zeitpunkt auch keinen weiteren anderen Staat) und auch der UN-Sicherheitsrat sah keinen Grund zur Legitimierung einer Intervention. Trotzdem führte die NATO einen Krieg gegen dieses Land. Das war der erste Aggressionskrieg, an dem sich Deutschland nach 1945 wieder direkt beteiligt hat. Der Kriegsgrund war ein bis heute zumindest umstrittener: die Verhinderung eines genozidalen Verbrechens. Die OSZE, damals mit einer Beobachtermission im Kosovo aktiv, konnte jedenfalls keine Hinweise liefern, die auf die Vorbereitung eines solchen Verbrechens schließen ließen. Viele *Beweise*, die damals präsentiert wurden, haben sich im Nachhinein als Fabrikate herausgestellt. Die NATO kennt viele Gefährdungen der Sicherheit. Das sind neben den bewaffneten Angriffen feindlicher Staaten auf das Territorium eines oder mehrerer Mitgliedsstaaten der Terrorangriff aus einem anderen Land, aber auch Unsicherheiten in der Versorgung mit Energie und Rohstoffen. Schließlich kommen noch Cyberangriffe hinzu.

Natürlich sind Cyberangriffe heute etwas anderes als die harmlosen Hackerscherze von einst. Mit dem Ausbau von Informationsnetzen sind unsere Infrastrukturen tatsächlich empfindlich angreifbar geworden. Aber wenn die NATO sich für alles zuständig glaubt, dann verschwimmt der Unterschied zwischen einem Computerwurm und einer Aggression. Schlimmer noch: Nun wirklich legale Vorgänge wie Handelsabkommen zwischen China und anderen Ländern mögen zu einer *Bedrohung* der ökonomischen Vormachtstellung des Westens führen, aber darin eine dem bewaffneten Angriff auch nur vergleichbare Bedrohung zu sehen, grenzt an Wahnsinn.

Könnte die Zukunft der NATO in einem Rückbau des Sicherheitsbegriffs liegen? Ja, das würde viel Druck von der NATO nehmen. Aber dennoch ist das unrealistisch. Solange die Vorstellung existiert, dass der Einsatz von militärischer Gewalt zur Sicherung ökonomischer Interessen berechtigt ist, wird die NATO ihre Existenz auch damit legitimieren wollen.

Dennoch ist die Idee eines Systems kollektiver Sicherheit richtig. Kollektive Sicherheit kann aber in Europa nicht funktionieren, wenn das größte europäische Land nicht Mitglied eines derartigen Systems ist, es vielmehr immer stärker zu konfrontativen Akten kommt. Auch der NATO-Russland-Rat hat sich als unzureichend erwiesen. Er taugte nur während Schön-Wetter-Perioden. Aber gerade während der Krim-Krise wurde er auf Eis gelegt, anstatt ihn zur Entschärfung der Lage zu nutzen. Aber auch vorher schon hatte Russland nichts davon. Solche Projekte wie der Raketenschutzschirm wurden trotzdem realisiert, trotz russischer Bedenken. Die Versicherung, dass dieser sich nicht gegen Russland richte, gilt die eigentlich immer noch? Natürlich mache ich mir keine Illusionen über die Schwierigkeiten eines Sicherheitsprojekts unter Einschluss Russlands. Die baltischen Länder, Polen und die Ukraine dürften skeptisch sein, ebenso Georgien. Nur welchen Weg soll es sonst geben?

Erste Schritte müssen in vertrauensbildenden Maßnahmen liegen: von Manöverbeobachtungen bis hin zu (Ab‑)Rüstungskontrollen. Aber schwieriger als einst die Bildung der Montanunion unter den ehemaligen Kriegsgegnern kann es auch nicht sein. Diese wurde nicht zuletzt deshalb gebildet, weil man wissen wollte, was sich in den damals wichtigsten Industrien, die kriegsrelevant sein können, so tut. Die heute noch immer pathetisch vorgetragene Rede von der EU als Friedensprojekt hat dort ihren historisch wahren Kern. In einem nächsten Schritt können Verträge über wechselseitigen Beistand das Sicherheitssystem vertiefen. Die NATO ist als Ausgangspunkt nicht geeignet. Das hat das Versagen des NATO-Russland-Rates gezeigt. Auch eine Art EU-NATO kann europäische Sicherheit nicht garantieren, einfach weil Russland draußen wäre. Dagegen könnte die OSZE einen Ausgangspunkt bilden. Das ist eine von vornherein inklusive Organisation: Alle Staaten Europas sind dabei. DIE LINKE betreibt jedenfalls nicht den Austritt aus der NATO. Das ließe die NATO in ihrem Charakter unverändert. Möglich wäre jedoch eine Einstellung der Mitwirkung in den militärischen Strukturen. Vorrang hat jedoch aus unserer Sicht das Zwei-Prozent-Ziel. Das ist, vergleicht man die Rüstungsausgaben allein der europäischen NATO-Staaten mit denen Russlands, eine Bedrohungsgeste gegenüber Russland. Langfristig strebt DIE LINKE jedoch die Auflösung der NATO an und ihre Ersetzung durch ein System kollektiver Sicherheit, wie ich es oben skizziert habe.

## Für eine solidarische Europäische Union

Zu den Positionen der LINKEN zur EU könnte man viel schreiben. Wenn sich die Europadebatte polarisiert, kommt es oft zu der zu verengten Bekenntnisfrage: Bist Du nun für oder gegen die Europäische Integration? Kein Mitglied der LINKEN, das ich kenne, ist gegen die Europäische Integration. Auch die politischen Forderungen, die im Einzelnen erhoben werden, ergeben nur dann überhaupt einen Sinn, wenn man die Partei DIE LINKE als proeuropäische Partei denkt. Das geht manchmal aufgrund der massiven Kritik, die wir üben, unter. Das Problem ist ein völlig anderes. Der Integrationsmodus der EU besteht im Kern darin, dass Integration als ökonomische Integration vorangetrieben wurde und andere Politikfelder darunter in Mitleidenschaft gezogen wurden. Es handelt sich um eine *negative* Integration. Negativ deshalb, weil der Rechtsbestand der jeweils nationalen Rechtssysteme, sofern sie der Herausbildung eines gemeinsamen Marktes mit der kompletten Bewegungsfreiheit des Kapitals entgegenstanden, entsprechend angepasst, also *negiert* wurde. Insbesondere da, wo es Standards wie soziale Rechte, einschließlich Arbeitnehmerrechte, und Verbraucherschutzrechte betrifft, hat das immer wieder Skepsis hervorgerufen.

Es wird daher Zeit für eine *positive* Integration. Diese muss einen Rahmen von rechtlich sicheren Standards umfassen, in denen Sozialstaatlichkeit, demokratische Mitbestimmung und ökologische Standards im Zentrum stehen. Das wird nicht gelingen, ohne neoliberale Doktrinen der Geld- und Sparpolitik aufzubrechen und deren Realisierung vor allem im Fiskalpakt zurückzunehmen.

Die Zurichtung des Südens der EU während der *Eurokrise*, die den Sozialstaat in diesen Ländern fast vollständig zerstört hat, und der Austritt Großbritanniens aus der EU, aber auch der Rückfall in nationale Abschottung zu Beginn der Corona-Krise verdeutlichen aus meiner Sicht, dass ein Kurswechsel geboten ist. Sonst könnte die EU scheitern. Gerade weil die EU ein Projekt ist, das Frieden zwischen den beteiligten Nationalstaaten gestiftet hat, ist sie es wert, um sie zu kämpfen.

## Internationale Zusammenarbeit

Die gegenwärtige Pandemie macht deutlich, dass es Ereignisse und Probleme gibt, die nichts mit Staatsgrenzen zu tun haben, für die es keine Staatsgrenzen gibt. Es ist zentral wichtig, den engen Horizont nationaler Egoismen zu überschreiten. Dabei gibt es *traditionelle* und *neue* Problemlagen. Als traditionell möchte ich die (aktuell zunehmende) globale Ungleichheit ansehen. Diese ist wesentlich das Ergebnis von Kolonialismus und anderen imperialen Politiken des Westens bzw. globalen Nordens. Daher existiert eine besondere Verantwortung des Nordens für die Beseitigung der globalen Ungleichheit. Als neue Problemlage kann man die Klimaentwicklung ansehen. Allerdings muss man den Unterschied zwischen traditionell und neu sofort wieder relativieren: Entwicklungsprojekte im globalen Süden sind vor allem da sinnvoll, wo sie den Pfad industrieller Entwicklung, wie durch den *Norden* vorgeprägt wurde, verlassen.

Oft, zu oft, hat die Entkolonialisierung die Wirtschaftsbeziehungen zwischen den (einstigen) Kolonien und ihren (einstigen) *Mutterländern* unangetastet gelassen. Rohstoffe und Halbfabrikate werden exportiert, die hauptsächliche Wertschöpfung findet dann jedoch in den importierenden Ländern des Nordens statt. Das wird als ungleiche* Terms of Trade* bezeichnet und gilt als Entwicklungshemmnis. Der Export vermag nicht als ausreichende Quelle für entwicklungsfördernde Binneninvestitionen zu dienen. Vielfach werden hier zu Recht Probleme benannt wie Korruption und ähnliche Phänomene, die wirksame Entwicklungspolitik erschweren. Aber die Grundprobleme ungleicher *Terms of Trade* blieben erhalten, selbst wenn *Good Governance*, das herrschende entwicklungspolitische Konzept, komplett realisiert würde. Dieses Konzept zielt letztlich auf reibungslosen Marktzugang, nicht jedoch auf die Behebung grundsätzlicher Fehlstellungen in den globalen ökonomischen Strukturen.

Allerdings gibt es eine Einschränkung. Eine ökonomische Entwicklung des Südens, die den Weg des Nordens einfach nur kopierte, letztlich also eine klimaschädliche Produktionsweise nur potenzierte, sollte vermieden werden. Das kann nur gelingen, wenn *grüne* technologische Innovationen kostengünstig transferiert werden können. Eine Möglichkeit besteht in der Vergabe von Lizenzen zu günstigen Konditionen. Das Thema Klima ist nun angeschnitten. DIE LINKE setzt sich für eine Umsetzung der Klimaziele des Pariser Abkommens ein. Allerdings halte ich die bisher favorisierte Herangehensweise, den CO2-Verbrauch zu verteuern, für vielleicht zielführend, aber mit unsozialen Nebenwirkungen verbunden und daher nicht für optimal. Für sozial Bessergestellte mag die Verteuerung von CO2 erträglich sein, für die Bezieher kleiner Einkommen, für Arbeitslose, für viele Rentner*innen und Bezieher*innen von Hartz IV ist das nur schwer oder überhaupt nicht zumutbar. Meine Partei schlägt daher für unterschiedliche Wirtschaftszweige und Produkte verpflichtende Ausstiegsziele vor. So wie es für die Kohleverstromung ein Ausstiegsziel gibt, so könnte das auch für Verbrennungsmotoren eingeführt werden etc.

Ein Thema, das eng mit der Europäischen Union und den Migrationsbewegungen verbunden ist, ist die Ernährungssouveränität. Durch den Export subventionierter Lebensmittel aus der EU ist die Landwirtschaft in vielen afrikanischen Ländern zerstört worden, was wiederum kleinbäuerliche Existenzen zerstört hat. Dadurch sind diese Länder, weil sie keine eigene Produktion mehr haben, auf den Import aus der EU angewiesen. Die zerstörte ökonomische Existenz wiederum ist eines der vielen Motive, das Land zu verlassen und anderswo ansässig zu werden. Durch den Klimawandel und die Ausbeutung von Bodenschätzen bewirkte weitere Zerstörung von landwirtschaftlich nutzbarer Fläche erhöht weiter den Migrationsdruck. Für die betroffenen Länder wäre der Wiederaufbau einer eigenen Landwirtschaft entscheidend, um so das Problem der Unterernährung zu entschärfen.

Das sind große Fragen, auf die schnell vielleicht keine Lösung gefunden werden kann. DIE LINKE fordert jedoch auch jenseits dessen, dass Deutschland sich endlich an seine Verpflichtungen bezüglich der ODA-Quote^2^ hält, also 0,7 % des Bruttonationaleinkommens für Entwicklungszusammenarbeit auszugeben. Zudem gibt es innerhalb der wissenschaftlichen Literatur zur Entwicklungszusammenarbeit die immer wiederkehrende Kritik, dass die ODA-Zahlen systematisch geschönt seien. In der Tat fallen darunter oft auch Projekte der zivil-militärischen Zusammenarbeit. Wenn ein alter LKW einer Entwicklungsorganisation überlassen wird, dann ist die *Ausgabe* natürlich eigentlich nicht vorhanden, es erfolgt eben nur eine fiktive Ausgabe. Zudem stehen zivil-militärische Zusammenarbeitsprojekte auch unter anderen Gesichtspunkten unter Kritik. So berichteten einige Entwicklungsorganisationen, dass die Sicherheit ihrer Projektmitarbeiter, aber auch die Akzeptanz ihrer Arbeit, eher abnehme, wenn sie eng mit dem Militär kooperieren. Sie werden dann oft als Anhängsel einer Besatzungsmacht wahrgenommen. Daher hat DIE LINKE immer eine kritische Haltung zur zivil-militärischen Zusammenarbeit gezeigt und deren Beendigung gefordert.

## Schlussbemerkung

Oft wird meiner Partei vorgeworfen, ihre Positionen zur Außen- und Europapolitik, insbesondere zur NATO, seien unrealistisch. Nun sind es aber gerade die *Realisten*, die eine Politik zu verantworten haben, die Destabilisierung und andere krisenhafte Entwicklungen sowie die Verstetigung von Unterentwicklung hervorgebracht haben. Ich würde daher mit einigem Stolz sagen, dass die Positionen meiner Partei nicht realistisch sind. Die Alternative dazu ist eine konkrete Utopie. Konkrete Schritte zu einer friedlichen Welt mit gerechter geteiltem Wohlstand können und müssen beschritten werden. Dafür stehen wir zur Verfügung.

## Footnotes

1 Sansano, G. H. (2020). «Verteidigung ist eine Gemeinschaftsaufgabe» – Interview. Neue Züricher Zeitung. https://www.nzz.ch/international/eberhard-zorn-verteidigung-ist-eine-gemeinschaftsaufgabe-ld.1589704. Zugegriffen: 1. Apr. 2021.

2 Official Development Assistance (ODA), welches den Anteil der öffentlichen Ausgaben für Entwicklungszusammenarbeit gemessen am Bruttonationaleinkommen meint.

